# Products of Lipid Peroxidation as a Factor in the Toxic Effect of Silver Nanoparticles

**DOI:** 10.3390/ma13112460

**Published:** 2020-05-28

**Authors:** Patrycja Paciorek, Mariusz Żuberek, Agnieszka Grzelak

**Affiliations:** Department of molecular Biophysics, Faculty of Biology and Environmental Protection, University of Lodz, Banacha 12/16, 90-237 Lodz, Poland; mariusz.zuberek@biol.uni.lodz.pl (M.Ż.); agnieszka.grzelak@biol.uni.lodz.pl (A.G.)

**Keywords:** oxidative stress, silver nanoparticles, lipid peroxidation

## Abstract

In our previous study we have shown that nanoparticles have different effects depending on the energy metabolism of the cell, which is an important factor in the context of oncology and diabetes. Here we assess the influence of AgNPs on cellular lipid components in varying glucose concentrations. To assess the effect of silver nanoparticles on cell lipids, we measured cell viability, the fluidity of the cell membranes, the content of amino groups in proteins, the level of lipid peroxidation products, the concentration of 4-hydroxynonenal (4-HNE), and the concentration of lipid peroxides. The obtained results show differences in the formation of lipid peroxidation products in cells exposed to oxidative stress induced by nanoparticles. In addition, we have shown that the metabolic state of the cell is a factor significantly affecting this process.

## 1. Introduction

While living in an aerobic environment, cells are exposed to the phenomenon of oxidative stress. The concept of stress and the mechanisms of the response to it were first described in 1936 [1] and the term “oxidative stress” was developed in the book of the same title in 1985. It was defined as an imbalance between oxidants and antioxidants, in favor of oxidants, that leads to interference in the transmission of signals and in damage to cellular structures [2].

Oxidative stress results in accumulated damage to DNA, proteins, and lipids, which can cause changes in cell metabolism. A high level of oxidative damage can even result in cell death. Cells have developed adaptive mechanisms to the conditions of a temporary increased level of oxygen free radicals and other reactive oxygen species which can be produced under the influence of various stimuli (e.g., nanoparticles, xenobiotics, radiation, toxins). Cell metabolism in normal, physiological conditions produces free oxygen radicals to serve as signal molecules activating intercellular and intracellular signal transduction pathways such as Nrf-2 or AP-1 [3,4].

Cells have the ability to make use of free radicals during normal metabolism. Likewise, they develop signal cascades to regulate oxidative stress, and they contain antioxidants to support the maintenance of normal redox status and prevent the induction of oxidative stress. Most importantly, they can delay or even inhibit the oxidation of cell-building molecules, even at low concentrations.

We can distinguish two classes among antioxidants. The first class includes enzymatic antioxidants, such as superoxide dismutase, glutathione peroxidase, and catalase. The second class consists of small, non-enzymatic antioxidants consumed with food, like ascorbate, β-carotene, or molecules synthesized endogenously, such as glutathione [2,5]. Some compounds can act as reactive oxygen and nitrogen scavengers despite their low reactivity with reactive oxygen species (ROS) while still playing an important role in cell protection due to their occurrence in high concentrations. This class of compounds includes proteins (ones that are not specialized antioxidative enzymes), peptides, and amino acids, among which tryptophan, tyrosine, histidine, and cysteine are the amino acids most sensitive to oxidation [5,6]. Misfolded proteins are shields to many molecules because they are most susceptible to oxidative modifications and after oxidation they undergo proteolysis via the proteasome pathway [7].

Under moderate oxidative stress conditions, activation of antioxidant defense systems take place mainly through induction of the nuclear factor Nrf2 (erythroid-derived 2) -like 2 and additionally by activation of NF-κB (nuclear factor kappa-light-chain-62 enhancer of activated B cells) and of mitogen-activated protein kinase (MAPK) cascades. Oxidative stress induced by nanoparticles which exceeds the adaptive capacity of the cell results in damage, mainly to mitochondrial membranes, causing irreversible damage to proteins, DNA, and lipids and committing the cell to apoptosis pathways [8,9,10]. 

Important secondary messengers in the process of adaptation/commitment to apoptosis are lipid peroxidation products [11]. Initially, the effects of lipid peroxidation were studied as damage to cell membranes and potential danger to their integrity. Not only are polyunsaturated fatty acids oxidatively damaged but also glycolipids, phospholipids, and sterols. The oxidation of lipids may occur via non-enzymatic or enzymatic pathways, the latter involving recruitment of such enzymes as lipoxygenases, cyclooxygenases, and cytochrome P-450. The metabolism of cells under aerobic conditions is associated with continuous lipid peroxidation. Under physiological conditions or under oxidative stress of a low intensity, lipid peroxidation products regulate pathways responsible for the antioxidant protection system, which makes the cells more resistant to further oxidative stress, while intensive lipid peroxidation can induce cell death by apoptosis, necrosis, or autophagy [12,13,14].

The most commonly studied secondary products of lipid peroxidation are malondialdehyde (MDA), propanal, hexanal, and 4-hydroxynonenal (4-HNE). MDA has been identified as being the most mutagenic, whereas 4-HNE is the most toxic peroxidation product [15]. 

4-HNE is presently the most intensively studied lipid peroxidation product and precise determination of its level is crucial for assessing the level of oxidative stress in a cell [16]. Its toxicity is associated with its high reactivity toward thiol and amino groups [17]. 

4-HNE is considered as the “second toxic messenger of free radicals [18,19], one of major generators of secondary oxidative stress” [19,20], “one of the major toxic products generated from lipid peroxides [19,21] one of the most physiologically active lipid peroxides [19,22]. It has been found that the level of 4-HNE regulates many transcription factors, e.g., nuclear factor (erythroid-derived 2)-like 2 (Nrf2) [23,24,25], activating protein-1 (AP-1) [26], nuclear factor kappa-light-chain-enhancer of activated B cells (NF-κB) [27], and peroxisome-proliferator-activated receptors (PPAR) [28,29]. It also activates pathways that are responding to stress, such as mitogen-activated protein kinases (MAPK) [30].

Intracellular production of oxygen free radicals stimulated under the influence of nanomaterials causes mobilization of the intracellular antioxidant defense system and restoration of intracellular redox balance [31]. Various kinds of nanoparticles cause oxidative stress, but metallic ones are best studied, especially those, like AgNPs, that may undergo Fenton-like reactions [32]. In the case of silver nanoparticles, the rich literature on the subject indicates that the toxic effect of those nanoparticles is related to their physicochemical nature, when the toxicity is compared to extracellularly administered silver ions [33]. Because of the common use of nanoparticles in various branches of industry they accumulate in the trophic chain and the abiotic environment. Nanoparticles accumulate in tissues and organs including the kidneys, brain, and liver.

The level of free radical production is strongly dependent on the energy metabolism of cells. Accordingly, in cells with limited activity of oxidative phosphorylation the production of reactive oxygen species is limited at lower glucose concentrations when compared to cells with increased respiratory chain activity. Although the production of ROS is elevated in the case of a higher activity of the respiratory chain, antioxidative defense mechanisms are induced, making the observable levels of ROS reduced. 

Diabetes is one of the diseases of civilization. It is associated with elevated glycemia, which makes it important to study nanomaterials in conditions that reflect physiological and pathological glucose concentrations.

In this work we present the effect of silver nanoparticles on the generation of lipid peroxidation products in Hep G2 cells (derived from hepatocellular carcinoma cells). We compare the cellular response to the toxic effects of silver nanoparticles in cells cultured in a medium with a physiologically normal glucose level and in cells cultured under conditions of the elevated glucose level that commonly occurs in hyperglycemia. Hep G2 cell line was selected for this because it originates from the liver and tolerates varying glucose availability levels.

## 2. Materials and Methods 

### 2.1. Cell Culture

Hep G2 cell line (ATCC, Manassas, VA, USA) was derived from hepatocellular carcinoma. Hep G2 cells were cultured in Dulbecco’s modified eagle medium (Thermo Fisher Scientific, Waltham, MA, USA) supplemented with 5.5 mmol/dm^3^ or 25 mmol/dm^3^ glucose. Both media were supplemented with 10% FBS (Thermo Fisher Scientific, Waltham, MA, USA). Cells were incubated in an atmosphere of 5% CO_2_ at 37 °C at 95% relative humidity. The passages were performed three times per week and the experiments were carried out on cells cultured for at least three weeks after transfer to a lower glucose medium.

### 2.2. Preparation of Silver Nanoparticles

Total of 4 mg silver nanoparticles of 20 nm nominal diameter (PlasmaChem, Berlin, Germany) were suspended in 1600 mm^3^ of MilliQ water and sonicated (4.2 kJ/cm^3^) with an ultrasonic homogenizer. Then, albumin (Sigma, Darmstadt, Germany) and concentrated PBS (Sigma, Darmstadt, Germany) were added to reach 2 mg/cm^3^ concentration of nanoparticles in PBS containing 1% albumin. 

### 2.3. Preparation of Cell Lysates

Cells were seeded in density of 1.3 × 10^6^ cell per flask (Nunclon, 25 cm^2^) and cultured in 5.5 and 25 mmol/dm^3^ glucose medium, were allowed to adhere for 24 h, and then were treated with 25 µg/cm^3^ silver nanoparticles and incubated for 24 h. Then the cell monolayer was trypsinized (1 cm^3^, 0.25%) (Sigma, Germany, Darmstadt), centrifuged at 100 relative centrifugal force (RCF) for 7 min, and tested for necrotic phenotype with trypan blue; the experiments were performed on cells with integral cell membranes. A pre-prepared lysis solution (water with protease inhibitors and EDTA (Thermo Fisher Scientific, Waltham, MA, USA) was added to the cell pellet to form the final cell density of 1 × 10^6^ cell per cm^3^. Lysates were prepared by freezing cell suspensions in ‒20 °C and were used in determination of amino groups in proteins, protein concentration measurement, and in evaluation of peroxides concentration.

Before each experiment, cell lysates were thawed and centrifuged at 10^4^ RCF for 10 min at 4 °C and the pellet was discarded.

### 2.4. Internalization of Nanoparticles

Hep G2 cells were seeded in 6-well plates (Nunclon, Thermo Fisher Scientific, Waltham, MA, USA) at a density of 5 × 10^5^ cells per well. After 24 h, silver nanoparticles were added to the cell culture at concentrations of: 50 μg/cm^3^, 25 μg/cm^3^, and 12.5 μg/cm^3^. After 4 h, the conditioned medium was discarded and the cell monolayer was released from the surface and fresh medium was added. The side scatter parameter was measured to assess the pace of AgNP accumulation (LSR2 flow cytometer). Areas under the curve were compared with Student’s t-test.

### 2.5. Cell Viability Measurement

Cell viability was estimated by assessing neutral red uptake on cells planted at a density of 10^4^ cells per well on 96-well plates (Nunclon, Thermo Fisher Scientific, Waltham, MA, USA). After 24 h of culture, H_2_O_2_ at concentrations (final) of 3.9 × 10^‒6^ to 10^‒2^ mol/dm^3^ was added to the cells for ten min. Then the cell cultures were rinsed with PBS (Sigma, Darmstadt, Germany) and fresh medium was added for 24 h. Then, cell cultures were supplemented with 50 µg/cm^3^ neutral red solution in a culture medium for 4 h, washed twice with a fixative (50% ethanol (Avantor Performance Materials Poland, Gliwice, Poland), 49% H_2_O (Milli-Q, Merck, Darmstadt, Germany), 1% acetic acid (Avantor Performance Materials Poland, Gliwice, Poland)), and absorbance was measured at a wavelength of 540 nm, as described previously [34]. The IC 50 values were compared with Student t-test, n = 6, α = 0.05.

### 2.6. Cell Membrane Fluidity Measurement

Cells were seeded at a density of 10^4^ cells per well on 96-well black plates (Nunclon, Thermo Fisher Scientific, Waltham, MA, USA). After 24 h, silver nanoparticles were added to a final concentration of 0.39–100 µg/cm^3^ for 4 and 24 h. The medium was removed and the cells were rinsed with HBSS (Thermo Fisher Scientific, Waltham, MA, USA) supplemented with 0.45% glucose. Fluidity measurement was made using pyrene. Pyrene solution dissolved in dimethyl sulfoxide (DMSO) in 20 µmol/dm^3^ concentration and an appropriate aliquot was added to Hanks’ balanced salt solution (HBSS) to achieve 20 µmol/dm^3^ pyrene concentration. This solution was added to the cell monolayers for 30 min. The fluorescence was measured at an excitation wavelength of 339 nm for monomer and excimer, and at an emission wavelength of 396 nm for monomer and 470 nm for excimer. Comparison of cells grown in medium with different glucose level was performed by comparing areas under the curves with Student’s t-test, n = 3, α = 0.05. Differences between varying concentrations of silver nanoparticles in the same medium were tested with anova followed by Dunnett‘s post hoc test.

### 2.7. Determination of the Content of Amino Groups in Proteins

The content of amino groups was determined with fluorescamine. Lysates (7.5 × 10^−2^ cm^3^) were added to each well on 96-well plates (Nunclon, Thermo Fisher Scientific, Waltham, MA, USA) followed by 2.5 × 10^−2^ cm^3^ of fluorescamine solution in dioxane (3 mg/cm^3^). The incubation lasted for half an hour. After this time, the fluorescence was measured at an excitation wavelength of 390 nm and an emission wavelength of 485 nm.

### 2.8. Protein Measurement

The protein content in the cell lysates was determined with Pierce 660 nm Protein Assay Reagent solution (Thermo Fisher Scientific, Waltham, MA, USA) according to the manufacturer’s instructions. The absorbance of the samples was determined at λ = 660 nm and the protein concentration was read from the standard curve. To prepare the standard curve, a bovine serum standard at a concentration of 2 mg/cm^3^ was used (Thermo Fisher Scientific, Waltham, MA, USA).

### 2.9. Determination of Lipid Peroxidation Products

Hep G2 cells were seeded on 6-well plates (Nunclon, Thermo Fisher Scientific, Waltham, MA, USA) at a density of 5 × 10^5^ cells per well. After 24 h, silver nanoparticles were added to the cell culture at concentrations of: 50 μg/cm^3^, 25 μg/cm^3^, and 12.5 μg/cm^3^. After 4 h, the conditioned medium was discarded, the cell monolayer was released from the surface, and fresh medium was added. BODIPY® 581/591 C11 (Thermo Fisher Scientific, Waltham, MA, USA) was used as the probe for lipid peroxidation products. It was added to the cell suspension at a concentration of 1 μmol/dm^3^, mixed and incubated for 30 min. Fluorescence was measured in the FITC and PE channels on flow cytometer (LSRII). Tert-butyl hydroperoxide was used as a positive control at a concentration of 2.5 mmol/dm^3^ to set up the assay.

### 2.10. Evaluation of the Level of 4-HNE in Cell Lysates

Evaluation of the amount of HNE produced in cells treated with silver nanoparticles was carried out using a commercially available kit (GenAsia, Shanghai, China), based on the ELISA method, in accordance with the manufacturer’s instructions. Statistical analysis was performed using Student’s t-test, n = 3, α = 0.05.

## 3. Results

All experiments were performed on cells grown in two variants of DMEM medium, supplemented with 5.5 mmol/dm^3^ glucose, and 25 mmol/dm^3^ glucose. All experiments included appropriate controls and were carried out on cells that were metabolically active. Statistical significance was tested at the level α =0.05.

### 3.1. Cell Viability Measurement

As we have shown in our previous work [34], the amount of glucose in the culture medium affects the redox status in Hep G2 cells. Cells grown in a medium at physiological glucose concentration (5.5 mmol/dm^3^) showed greater resistance to oxidative stress than cells grown in medium with increased glucose concentration, which manifested itself as increased viability under AgNP treatment. Oxidative stress, being a major factor in the toxicity of AgNPs, was described in numerous papers, including experiments where AgNPs-induced ROS were quenched, which in turn alleviated the toxic effects of AgNPs [35].

**Figure 1:**
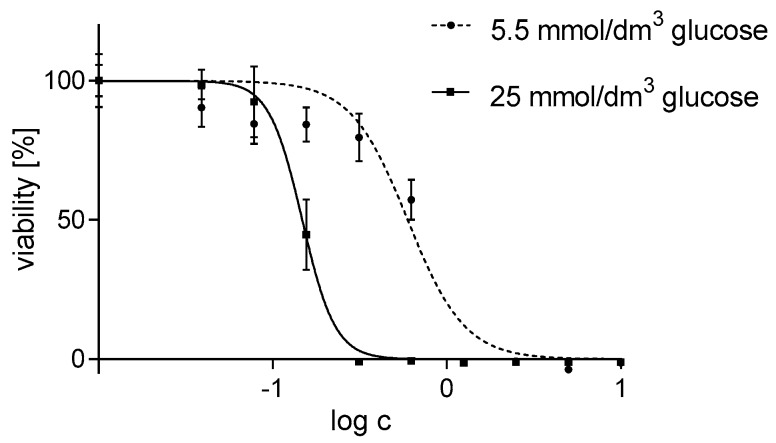
The effect of hydrogen peroxide on the survival of Hep G2 cells (1 × 10^3^ cells per well). The cells were cultured in a medium with two variants of glucose concentration: 5.5 mmol/dm^3^ and 25 mmol/dm^3^ for 24 h, treated with H_2_O_2_ for 10 min, the measurements were taken after another 24 h. IC 50 parameters were compared with Student’s t-test, n = 6, α = 0.05.

To verify this hypothesis, we studied the effect of exogenous hydrogen peroxide on Hep G2 cells grown in different glucose concentrations. We have shown that the viability of cells exposed to the same hydrogen peroxide concentration is four times higher when cells are grown in a medium containing 5.5 mmol/dm^3^ glucose compared with cells grown in 25 mmol/dm^3^ glucose (Figure 1). These IC50 values are similar to those obtained for the same cell line grown under the same condition but exposed to silver nanoparticles. 

### 3.2. Lipid Peroxidation Products

Peroxide radicals were measured after exposure to silver nanoparticles in order to determine the initiation of lipid peroxidation. In this experiment, cells were grown in different glucose concentrations and incubated with nanoparticles for 4 h. Lipid radicals were detected by a BODIPY ™ 581/591 C11 probe (Thermo Fisher Scientific, Waltham, MA, USA); fluorescence was measured in a flow cytometer.

The level of peroxide radicals was estimated after 4 and 24 h incubation with AgNPs. Previous experiments have shown that the first signs of oxidative stress (after two h exposure) to AgNPs are noticeable by the increased level of H_2_O_2_ and superoxide anion radical secretion in Hep G2 cells, whereas after 24 h cells mount an antioxidant defense [36].

**Figure 2:**
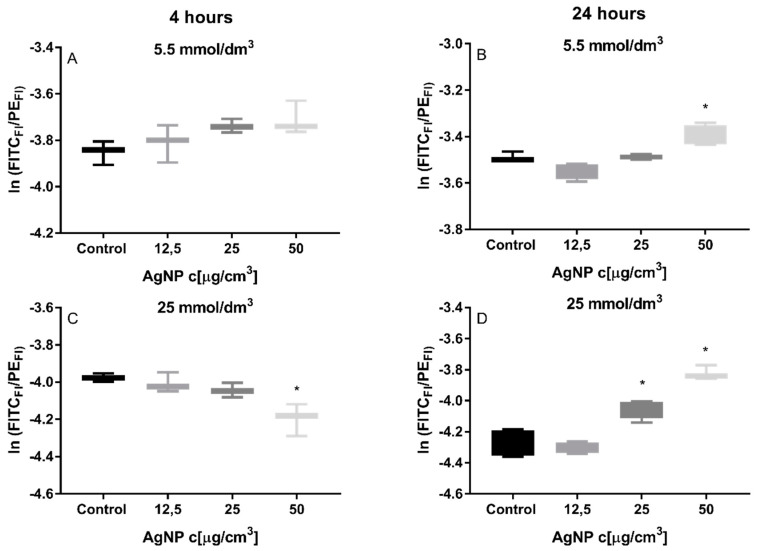
Generation of lipid peroxides in Hep G2 cell line grown in 5.5 (**A**,**B**) and 25 mmol/dm^3^ (**C**,**D**) glucose media, exposed to silver nanoparticles for 4 (**A**,**C**) and 24 h (**B**,**D**). Cells were seeded in density of 5 × 10^5^ per well and lipid peroxides were analyzed with flow cytometry (BODIPY® 581/591 C11). * denotes statistically significant difference compared to control (untreated cells). ANOVA followed by Dunnett’s post hoc test, n = 6, α = 0.05.

The intensity of lipid peroxidation induced by ROS is shown in Figure 2. To assess this parameter we used a BODIPY ™ 581/591 C11 probe, whose fluorescence maximum after oxidation shifts from λ = 590 nm to λ = 510 nm. After cytometric analysis, the results were described as the quotient of the maximum fluorescence from the FIT-C channel to the maximum fluorescence from the PE channel. 

After a 4h incubation with silver nanoparticles, no increased production of oxidized lipid species was observed in cells grown at the physiological glucose level, whereas in the case of cells grown on higher glucose level in the medium, a statistically significant decrease in oxidized lipids was observed in cells incubated with 50 μg/cm^3^ nanoparticles, thus showing that an antioxidant response took place in cells cultured in 25 mmol/dm^3^ glucose and exposed to a high concentration of silver nanoparticles.

An increased amount of lipid peroxides was observed in cells grown under conditions of elevated glucose concentration incubated for 24 h with 25 μg/cm^3^ and 50 μg/cm^3^ silver nanoparticles. After the same exposure time for cells grown in a medium with physiological concentration of glucose, a statistically significant increase in lipid oxidation products was observed, though only in samples incubated with silver nanoparticles at a concentration of 50 μg/cm^3^. Antioxidant defense mechanisms after prolonged incubation with silver nanoparticles worked with greater efficiency in cells grown in a medium with a physiological concentration of glucose compared to cells cultured at a high glucose concentration.

### 3.3. SSC-A

We examined the SSC-A parameter in order to verify whether the pace at which silver particles accumulate is the same in both growth conditions of the Hep G2 cell line.

**Figure 3:**
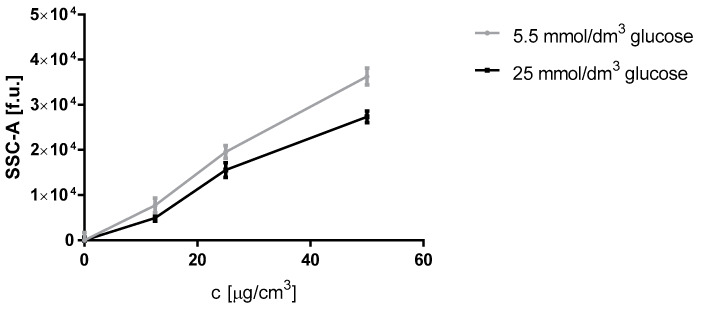
Measurement of the SSC-A parameter in Hep G2 cells (5 × 10^5^ cells per well) grown in 5.5 mmol/ dm^3^, and 25 mmol/ dm^3^ glucose media after 4 and h incubation with 12.5, 25, and 50 μg/cm^3^ silver nanoparticles concentrations. Comparisons between cells cultured in media with different glucose concentration was determined by comparison of areas under the curves with Student’s t-test.

The evaluation of nanoparticle accumulation in Hep G2 cells was made by means of the SSC-A parameter analysis. It is a method recognized in the literature for assessing the accumulation of nanoparticles in cells [37]. We observed an accumulation of nanoparticles in cells after 4 h incubation, and we demonstrated that the amount of nanoparticles accumulated intracellularly is proportional to the concentration of nanoparticles in the medium and that a higher accumulation took place in cells cultured in a medium with a physiological glucose concentration (Figure 3). 

### 3.4. Measurement of Lateral Membrane Diffusion

Cell membrane is composed of lipids and proteins that form together a compact structure permeable only to selected molecules. The free radicals formed during oxidative stress disturb the membrane structure, damaging it and reducing its fluidity. Not only are polyunsaturated fatty acids damaged but also glycolipids, phospholipids, and sterols [38]. 

We assessed the changes in membrane lateral diffusion of pyrene under silver nanoparticle treatment.

Pyrene is a probe that locates in the membrane as a monomer or an excimer, and its form depends on the viscosity of the microenvironment. The pyrene monomer and pyrene excimer have different fluorescence emission wavelengths. The membrane lateral diffusion coefficient is a function of the ratio fluorescence intensity of pyrene excimers and monomers.

**Figure 4:**
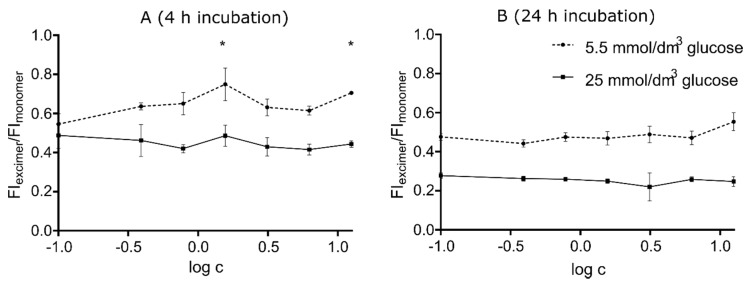
Alterations in the fluidity of the membrane in cells exposed to different silver nanoparticle concentrations were estimated by the ratio of fluorescence intensity of pyrene excimer and monomer. Cells (1 × 10^4^ per well) were cultured in 5.5 mmol/dm^3^ and 25 mmol/dm^3^ glucose concentration medium and incubated with silver nanoparticles for 4 (**A**) and 24 (**B**) h. AgNPs were used in concentrations: 0.39, 0.78, 1.56, 3.125, 6.25, and 12.5 µg/cm^3^, concentrations were logarithmized for graphing purposes. Comparison of cells grown in medium with different glucose levels was performed by comparing areas under the curves with Student’s t-test, n = 3, α = 0.05. Differences between varying concentrations of silver nanoparticles in the same medium were tested with anova followed by Dunnett’s post hoc test and marked with *.

Comparative analysis of the curves showed that the membrane lipids lateral mobility, both at 4 and 24 h incubation (Figure 4A,B), was lower in cells grown in high-glucose medium than in regular medium.

We noticed a statistically significant increase in lateral diffusion correlating with the increasing dose of silver nanoparticles only in the membranes of cells cultured in a medium with a physiological glucose concentration incubated with nanoparticles for 4 h (Figure 4A)

Differences in lateral diffusion of cells grown at different glucose concentrations might be due to a different response to nanoparticles present in the cell environment.

### 3.5. HNE Concentration as One of the Final Products of Lipid Peroxidation

HNE is one of the secondary products of lipid peroxidation, and its increased concentration occurs under oxidative stress conditions. HNE is a commonly used marker of the intensity of lipid peroxidation.

**Figure 5:**
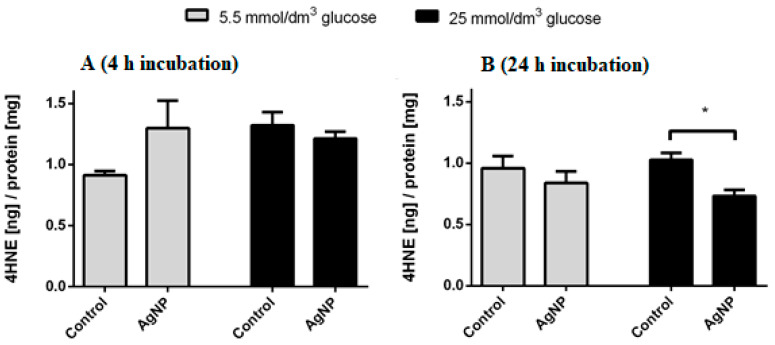
HNE content of cells cultured at different glucose concentrations (ELISA). The cells were subjected to a 4 h (**A**) and 24 h (**B**) treatment with silver nanoparticles (12.5 µg/cm^3^), untreated cells performed as the control; * denotes a statistically significant difference with respect to the control, Student’s t-test, n = 3, α = 0.05.

The amount of formed HNE was determined using the ELISA method with a commercially available kit. Both cell cultures grown in medium with increased glucose concentration and at physiological glucose concentration showed no statistically significant differences in the HNE content after 4 h incubation with silver nanoparticles, (Figure 5A). After extension of incubation up to 24 h (Figure 5B), cells grown in medium with elevated glucose showed a reduction in HNE content compared to cells that were not treated with silver nanoparticles, whereas cells grown in medium with low glucose concentration that were incubated with silver did not show statistically significant differences in HNE when compared to control cells.

### 3.6. Amine Group Level in Proteins

Amine groups are found in both proteins and lipids. In proteins, they are a functional fragment of N-terminal and basic amino acids, and in lipids they form a hydrophilic head due to their polar nature.

Amine groups react with carbonyl groups of HNE and other aldehydes. This reaction results in the formation of Schiff base and Michael adducts [17], which reduces the pool of free amino groups.

To assess whether the incubation of Hep G2 cells results in a loss of amino groups, we estimated the amine group level in proteins in cells after exposure to nanoparticles, using a fluorescent probe.

**Figure 6:**
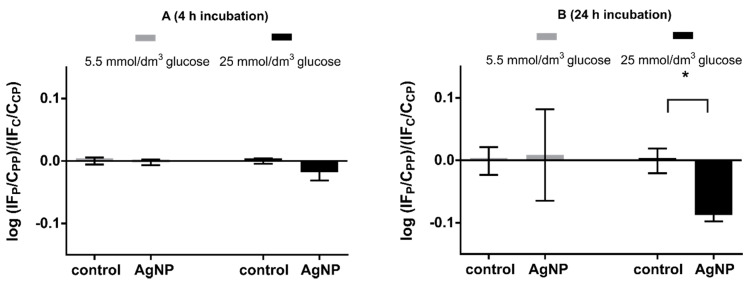
Logarithm of the fluorescence intensity ratio of protein-bound fluorescamine for cells exposed and nonexposed to nanoparticles (control). Fluorescence was normalized with respect to the protein content. Statistical analysis was performed using Student’s t-test, n = 3. The concentration of silver nanoparticles was 25 μg/cm^3^. Cells were collected after 4 h incubation (**A**) and after 24 h incubation, (**B**) with silver nanoparticles. * denotes a statisticcally significant difference.

In the case of the 4 h incubation of Hep G2 cells with silver nanoparticles, cells cultured both at physiological and increased glucose concentration did not show statistically significant differences in the level of protein amine groups with respect to control (Figure 6A). After 24 h of incubation with silver nanoparticles, a decrease in the amount of amino groups was observed in cells grown at elevated glucose conditions. Such differences were not noted in cells cultured at physiological glucose concentration (Figure 6B). This result may indicate that the level of oxidized amino groups increases in time in cells grown in an environment with an increased level of glucose when treated with silver nanoparticles. On the other hand, it can be assumed that cells grown in an environment of natural glucose concentration have developed mechanisms of long-term defense against amino groups oxidation.

## 4. Discussion

The adaptation mechanism of Hep G2 cells to stress conditions has been already described [34]. The toxicity of AgNPs has been demonstrated to be different under both cell culture conditions and dependent on the redox status of the cells. The presented experiments, in which hydrogen peroxide was used as an exogenous factor inducing oxidative stress, confirmed that cells cultured in 5.5 mmol/dm^3^ glucose medium are characterized by an increased resistance to the toxicity of the oxidant compared to cells grown at increased glucose concentration (a three-fold change in the IC50 parameter). This disparity in the response of cells cultured in different media leads us to conclusion that ROS play a key role in nanoparticle toxicity.

ROS generated during the incubation of cells with AgNPs are initiators of free radical processes e.g., lipid peroxidation or DNA damage. The superoxide anion is the primary free radical produced as a result of the activity of AgNPs. In our previous studies we attempted to resolve the sequence of events that are responsible for the toxicity of nanomaterials. We also determined the exposure time after which ROS secondary to superoxide anion appear as a consequence of the action of AgNPs [34].

We employed 4 and 24 h incubation with AgNPs. The 4 h incubation allowed us to measure the chosen parameters in cells exposed to AgNPs for a short time, whereas 24 h incubation allowed us to analyze the fraction of surviving cells, and consequently, evaluate the alterations in non-damaged cells.

There is wide range of lipid categories and they all are heterogenous when it comes to their structure and functions served. Their multiple roles make them crucial cell components. Being triglycerides, lipids are energy storage molecules and their hydrolysis releases energy contributing to thermoregulation in adipose tissue. As phospholipids, they are main components of cell membranes and define its unique properties. In the form of sphingolipids and steroid hormones (estrogens, testosterone) they participate in the regulation of intracellular transmission pathways by activating receptors specific for themselves, or receptors dependent on G proteins. Moreover, lipids support the catalytic activity of some enzymes, and they are also substrates in the synthesis of phospholipids and prostaglandins [39].

What lipids have in common is their lipophilic character, which means they are highly soluble in non-polar solvents. Chemical agents (spectrum of xenobiotics such as pesticides and nanomaterials) or physical factors (high-energy electromagnetic radiation, ultrasounds) cause lipids to undergo free radical reactions. Substances able to regulate the intracellular metabolism may be formed as a consequence of such oxidation reactions. Intracellular transmission pathways can be triggered by activating repair pathways in conditions where there are a moderate amount of oxidized lipids, however, in the case of increased oxidative stress and a large amount of oxidized lipids, cells may be directed to apoptotic pathways, or in extreme cases, it can lead to necrotic death [40].

As we mentioned before, lipid peroxidation is a process taking place in physiological conditions as a consequence of normal metabolism. A complex mixture of products that are difficult to analyze are formed in lipid peroxidation reactions. The problematic mixture of oxidative lipids derives from the complex and cyclical nature of the peroxidation process. To simplify the lipid peroxidation reaction we may present it in four stages: (i) The lipid hydroperoxide (LOOH) formation stage, (ii) the lipid free radical (L *) formation stage, (iii) the breakdown of lipid hydroperoxides into secondary oxidation products, and (iv) the formation of tertiary oxidation products. Each of these stages depends on a number of factors, such as the concentration of oxygen, or the presence of heavy metals (copper, iron, manganese) and antioxidants. All these processes run simultaneously, which allows unstable peroxidation products to react with themselves and with other non-lipid components of cells. This can perpetuate oxidative stress through the escalation of the formation of free oxygen radicals, or the regeneration of lipid radicals (L *). The peroxidation process is best described for unsaturated fatty acids, as not all lipids in mammalian cells undergo this process.

Oxidative stress, being the initiating factor of lipid peroxidation, is a paradigm that prompted us to monitor lipid peroxidation products after 4 and 24 h exposure to nanoparticles. The observations gave us an understanding of lipid peroxidation products formed during short-term exposure, and of changes in the parameters associated with lipid peroxidation in cells that survived 24 h incubation with nanoparticles. All experiments were carried out on metabolically active cells. The first experiment, assessing oxidative stress, was carried out with flow cytofluorimetry using fluorescent probes specific for individual free radicals and reactive oxygen species. All experiments included appropriate cytometric controls (cells not treated with a stress-initiating factor, stained with a probe and not stained with a probe, and positive controls where it was possible to perform such a control).

While analyzing the SSCA (Figure 3), we observed that a larger amount of nanoparticles penetrates into cells cultured on a medium supplemented with glucose at a concentration found in the blood plasma of healthy subjects more easily than into cells cultured on a higher glucose concentration. In both variants, the experiments were performed with the same dose of AgNPs. The experiments were performed at 80% cell confluence in each case. This type of calculation is important, especially when testing the toxicity of NP suspensions, as NPs do not form real solutions and the factor limiting their effect is the ratio of the quantity of NPs to the cell surface.

Despite the fact that NP loading is more efficient in cells cultured in a medium containing 5.5 mmol/dm^3^ glucose concentration, the biological effect of nanoparticles, i.e., the limitation of cell survival is lower. Cells grown at physiological glucose concentrations and adapted to continuous physiological oxidative stress caused by oxidative phosphorylation carried out in mitochondria, have a high redox capacity and are able to tolerate higher amounts of AgNPs.

Cell membrane fluidity is an important parameter characterizing the condition of the membrane. The cell membrane is a dynamic and not homogeneous structure. Cell membranes are mainly built of three classes of lipids (glycerophospholipids, sphingolipids, and sterols), which do not form a homogeneous lipid environment but heterogeneous microdomains—so-called lipid rafts—that are rich in cholesterol and glycosphingolipids. These structures have been discovered in many cell types. They are located both in the plasma membrane and in intracellular membranes [41]. The membrane’s properties are determined by the components of the lipid rafts. Glycosphingolipids are responsible for reducing the fluidity of the membranes, and cholesterol is involved in maintaining its stiffness. A small amount of phospholipids also contributes to building these rafts, although they are more saturated than the surrounding lipids. As a result of that, glycosphingolipids are packed differently and the rafts are isolated as separate domains in the membrane. These properties cause differences in the density of rafts and their lipid surroundings. Rafts are tightly packed domains as opposed to other regions of the membrane. Within the rafts, cholesterol proteins and sphingolipids interact with each other. The functionality of cell membrane domains is determined by the protein component. Some of them are caveolins, Src family of kinases, GPI anchored proteins, MAPK, EGF, PDFG, or CD55 [42,43]. Proteins interact with the membrane by binding to cholesterol (caveolin, integral membrane protein) [44] or by myristoylation and palmitoylation (e.g., eNOS) [45]. There is also a group of proteins that temporarily associate to lipid rafts after activation [46]. Also the lipid composition of the membrane affects its fluidity experiments have shown that a cholesterol increase in hepatic microsomes caused decreased fluidity of the membrane and in vitro modification of the cholesterol content of rat liver microsomes [47]. 

Erythrocytes are most commonly used in membrane fluidity studies. Because of their simple structure and very well characterized cytoskeleton-lipids interactions, erythrocytes are a convenient model for studying the parameters of lipid components. The basic methods of studying membrane fluidity employ fluorescent markers and electron paramagnetic resonance spectroscopy. In fluorimetric methods, pyrene is used, and the fluorescence of the probe is assessed after its incorporation into the cell membrane, or TMA-DPH is employed and fluorescence anisotropy is assessed [48]. However, in studies using EPR, the signal of specifically labeled fatty acids or the phospholipids that build into the cell membrane is analyzed [49].

In nanoparticle-toxicity studies the effect of pH on cell membrane fluidity is frequently neglected. During cell growth, subtle changes in the pH of the cell medium occur. The effect of pH on the fluidity of erythrocyte membranes has been proven [50].

We used pyrene in our study, a probe that builds into the plasma membrane. The experiments were conducted on cells grown with different glucose availability and cultured for a maximum of 48 h without changing the medium. Because the plasma membrane separates the cell from the external environment, it is also the first site affected by the environment. Despite its effect on membrane fluidity, pH is an often neglected parameter. In the paper by Yamaguchi T et al. [50], the effect of pH on the fluidity of the erythrocyte membranes was proved. Nitroxide derivatives of fatty acids labeled at the depths 5C and 12C were used to assess membrane fluidity by the EPR technique. In the tested pH range (3-9,1) no changes in membrane fluidity were detected at the depth of C5, while at the depth of 12C changes in fluidity were noticed throughout the entire range of pH studied. The authors explained the changes in lipid fluidity as being the consequence of changes in the conformation of proteins. Furthermore, experiments in which the erythrocytes were depleted and enriched in cholesterol were carried out These proved that cholesterol was important for the fluidity of the membranes at low pH, while at high pH such an effect was not observed. In the case of eukaryotic cells grown under controlled pH conditions, this mechanism does not seem to have great importance, however, but it is essential to remember that pH alteration is a factor which can affect cellular homeostasis. It has been proven that a pH decrease to 7.0 causes a significant membrane fluidity increase, resulting in its reduced permeability [51]. There are also reports suggesting that pH alteration is a factor that strongly affects the fluidity of the plasma membrane. Other authors [48] reported that by incubating blood cells from healthy people and placing them in an incubation buffer containing elevated glucose level, TMA-DPH-fluorescence anisotropy was increased, evidencing a reduction in membrane fluidity of the blood cells studied. In that study, the level of glycation of hemoglobin and membrane proteins was also evaluated. A relationship between the level of glycation and the duration of the experiment was found. Moreover, the authors observed that short incubation times for blood cells with glucose caused a progressive decrease in membrane fluidity over time, until a plateau was achieved for all tested parameters, which indicates a new equilibrium state was attained in the studied systems [48].

In our work, we observed the effect of nanoparticles on membrane fluidity in relation to the concentration of nanoparticles (0.8–12.5 μg/cm^3^) and time of incubation (4 and 24 h). High concentrations of silver nanoparticles (above 12.5 μg/cm^3^, data not shown) caused high disturbance in the fluorescence signal, making reliable assessment of the fluidity impossible to conduct.

We observed increased membrane fluidity in cells grown in a medium containing the lower concentration of glucose after a short time exposure to nanoparticles, while in medium with elevated glucose concentration no changes in this parameter were noticed. This may point to the stabilizing effect of glucose on the Hep G2 cell membrane. A similar effect was observed by Lemos GS et al. [52] who found that erythrocytes were more resistant to hemolysis in the presence of 0.9 g/dl NaCl and glucose, indicating a stabilizing effect of glucose on red cell membranes [52].

Endocytosis is the main mechanism by which nanoparticles penetrate cells. It takes place in cholesterol-rich areas of the membrane, such as lipid rafts [53]. Therefore, nanoparticles may be able to change cell membrane fluidity by depleting it of cholesterol. Analysis of the SSC-A parameter showed an increased silver nanoparticle loading in cells cultured in a medium with a physiological glucose concentration compared to cells grown in a medium with increased glucose concentration (Figure 3).

Based on the conclusions of Yoshida et al. and our own data, we suggest a mechanism which can explain the differences in the rate of nanoparticle penetration into cells. In cells whose metabolism is based on glycolysis, large amounts of methylglyoxal (a by-product of glycolysis) is formed due to the high glucose content in the culture medium [34]. It was found that in cells treated with methylglyoxal, glucose supplementation was reduced by limiting the activity of glucose transporters GLUT1 and GLUT4. This is because methylglyoxal inhibits the activity of the transporters, and its prolonged presence leads to endocytic changes. It was also found that the internalization of transporters in cells stimulated with methylglyoxal was slower than in control cells [54].

We proved that in Hep G2 cells grown in a medium with increased glucose concentration the penetration of silver nanoparticles is less efficient than in cells grown in physiological glucose concentration (Figure 3). This may be caused by reduced endocytosis which occurred because of increased formation of methylglyoxal.

Moreover, on the basis of comparative analysis of SSC-A and membrane fluidity experiments, it may be suggested that the change in fluidity is associated with membrane depletion in cholesterol caused by penetration of silver nanoparticles into the cells via endocytosis that occurs in cholesterol-rich areas. Intensive endocytosis may deplete the membrane of cholesterol, causing an increase in the fluidity of the cell membrane.

As we have shown in our previous work [55], the presence of elevated glucose in culture medium caused an increase in observable ROS levels. It is possible that reactive oxygen species not compensated by increased antioxidant activity will result in elevated levels of lipid peroxidation. In turn, oxidized components of the cell membrane can manifest themselves as a reduced fluidity of cell membrane and this condition will promote decrease in penetration of silver nanoparticles through the membrane. The decrease in fluidity can be caused by a change in the amount of PUFA and cholesterol in the membrane of cells cultured on high glucose medium.

We used a BODIPY 581/591 C11 probe to estimate the amount of free lipid radicals detected. The oxidation of the lipid component (Figure 2) did not affect the membrane fluidity of the examined cells. This experiment showed that the amount of oxidized lipids did not change in both cell culture variants, irrespective of the incubation time with AgNPs at a concentration of 12.5 μg/cm^3^ and less. Perhaps interactions of the oxidized proteins with the cell membrane are much more important for cell membrane fluidity changes in Hep G2 cells than direct oxidation of membrane lipids, as in the erythrocyte model.

Our previous publication has shown that endocytosis causes the increased appearance of oxysterols in the cytosol, which is a result of the actions of free oxygen radicals and the occurrence of the increased level of cholesterol-rich endocytic vesicles inside the cells. The appearance of oxysterols activates the action of the LXR transcription factor, which is important in maintaining intracellular sterols homeostasis. Regulation through LXR with oxysterol participation may be one of the mechanisms responsible for cell resistance to the toxic effects of silver nanoparticles, the more so that a similar biological effect has been observed on two cell lines of different tissue origin [56].

Figure 5 presents the HNE level in cell lysates. 4-HNE is not only a toxic lipid peroxidation product but also an important signaling molecule. Its appearance in the intracellular environment is an oxidative stress marker. The broad spectrum of interactions of 4-HNE with cellular components may be the signal that directs the cell to the pathway of survival or programmed death, depending on the amount of 4-HNE.

Lipid peroxidation products (4-HNE, MDA, acrolein, and crotonaldehyde) can form adducts with DNA. For the first time, such adducts were observed in cells of adipose tissue. The rate of adduct formation with guanosine varies, and it decreases with the length of the carbon chain of lipid peroxidation products. The fastest adduct formation was observed for acrolein and the slowest for 4-HNE. Propane products of lipid peroxidation can be oxidatively modified to epoxy-aldehydes and form etheno- adducts with DNA. It has been found that 4-HNE-DNA adducts increase the frequency of mutations and inhibit DNA synthesis [57].

In the liver, lipid peroxidation products prevent the activation of NF-κB and TNF expression [58]. This action is based on the phosphorylation of the inhibition NF-κB by HNE’. In this scenario, NFκB cannot be activated and enters the proteolysis pathway. In addition, 4-HNE prevents NF-κB translocation into the cell nucleus, thereby reducing the cellular possibility of defense against oxidative stress [59].

There are studies showing that HNE toxicity is modulated by the level of glutathione transferases [60]. Glutathione transferases play an important role in signal transduction, with regard to their involvement in cell survival and apoptosis pathways. Inhibition of JNK protects tumor cells from apoptosis. The complex may dissociate and JNK can phosphorylate c-Jun, which is composed of AP-1. As a result, AP-1 targets are induced and the process leads, inter alia, to cell death [61]. One JNK activator is HNE. 4-HNE triggers JNK to cause the N-terminal fragment of c-Jun, binding and phosphorylating two sites within the activation domain of this transcription factor. As a result, c-Jun forms a complex with AP-1 which activates caspase-3, starting apoptosis [62].

Our experiments proved that the toxic effect of nanoparticles strongly depends on the glucose concentration in the culture medium. Analysis of gene expression related to oxidative stress showed that one of the most intensively regulated enzymes are glutathione transferases [34]. In cells grown at low glucose concentrations, gene expression of glutathione transferases was increased, as opposed to the expression in cells grown on a medium with a higher glucose concentration. This dependency may explain why cells grown in a lower glucose concentration show a higher resistance to the oxidative stress induced by silver nanoparticles.

Another HNE function is to regulate UCP proteins. UCP proteins are found in the internal mitochondrial membrane of all eukaryotes [63,64]. They regulate the efficiency of oxidative phosphorylation. Their role is to reduce mitochondrial reactive oxygen species (ROS) production by dissipating the proton gradient across the inner mitochondrial membrane, and thus to protect cells from oxidative stress [65]. ATP synthase activity depends on the proton gradient. In conditions of the absence of free ADP, ATP synthase does not ensure the return of protons to the matrix. It leads to the accumulation of H+ in the inner mitochondrial space. Too high a ΔμH + may promote the intensification of side reactions between molecules that are reactive towards each other—such as semiquinones and molecular oxygen—resulting in excessive ROS formation [66]. It was found that uncoupling protein (UCP) activity can be stimulated by lipid peroxidation products (e.g., HNE), derived from the elevated level of the superoxide radical anion. One of the UCP proteins found in the liver is the UCP2 protein. Although its level in mature hepatocytes is relatively low, it increases in conditions of intensified oxidative stress [67].

This is one of the mechanisms responsible for the greater resistance of Hep G2 cells grown in a physiological glucose medium to oxidative stress induced by nanoparticles. UCP2 limits the oxidative stress associated with the activity of the mitochondrial chain in which HNE may contribute. Increased UCP2 concentration in the oxidative stress condition that is causing the HNE intensified formation may be the mechanism that leads to the observed HNE depletion in cells cultured in the medium with high glucose concentration and incubated with AgNPs for 24 h. This ensures protection of the cells against reactive oxygen species produced as a result of the action of nanoparticles.

As we have already mentioned, HNE can bind to amine groups and form so-called Schiff-bases and Michael adducts. It is predominantly Michael adducts that appear in the cell. Schiff-bases are more difficult to detect due to the reversibility of the reaction in which they are formed. HNE has been shown to bind to cytochrome c oxidase and form Michael adducts [68]. Cytochrome c oxidase is the terminal protein of the electron transport chain in the mitochondrion. Inhibition of this protein prevents electrons transmitted in the electron transport chain being transferred to the oxygen molecule. This phenomenon does not only inhibit the process of aerobic respiration, but also causes accumulation of electrons in the inner space of the mitochondrion. The longer it continues, the greater the chance that non-specific reduction will occur. It increases the probability that the oxygen molecule will not be reduced to water and that superoxide anions will be produced by a one-electron reduction. In this way, HNE can contribute to the perpetuation of oxidative stress. We presented changes in the level of protein NH_2_ groups in Figure 6. The decrease in amino residues in cells cultured in a high glucose medium incubated with silver nanoparticles for 24 h was the only statistically significant change. This may suggest formation of adducts with molecules that were originally NH_2_ groups. As previously discussed, HNE is an important signaling molecule in cells. It is possible that despite the decrease of HNE formation (Figure 5), HNE present in cells cultured in a medium with high glucose concentration under the influence of silver nanoparticles is directed to the mitochondrion so as to stop the respiratory chain.
